# The evaluation of normal ocular parameters in two breeds of hedgehogs

**DOI:** 10.1002/vms3.1055

**Published:** 2023-02-15

**Authors:** Ghazal Aftab, Farnoosh Arfaee, Ahmad Asghari, Taghi Zahraei Salehi

**Affiliations:** ^1^ Department of Clinical Sciences Science and Research Branch Islamic Azad University Tehran Iran; ^2^ Faculty of Veterinary Medicine Department of Microbiology University of Tehran Tehran Iran

**Keywords:** conjunctival flora, fundoscopy, Hedgehog, intraocular pressure, phenol red thread test, Schirmer tear test

## Abstract

**Objectives:**

The purpose of this study was to evaluate conjunctival microflora and measure normal tear production and intraocular pressure (IOP) in two breeds of hedgehogs (long‐eared hedgehogs and Brandt's hedgehogs).

**Methods:**

Forty‐eight hedgehogs from two different breeds were chosen for the study. Tear production was measured using the Schirmer tear test (STT) and phenol red thread test (PRTT) in both eyes. IOP was measured using a rebound tonometer. To perform microbiological sampling one drop of tetracaine was instilled in the eyes. Two sterile microswabs were used to collect samples for the microbial and fungal culture. All the microswab samples were transferred in phosphate‐buffered saline (PBS) to the laboratory for culture. Two MacConkey and two blood agar media plates were employed for each eye. Oneplate of sabouraud dextrose agar (SDA) was used for the fungal culture for each eye. Standard biochemical tests were performed to identify the isolated organisms.

**Results:**

The mean STT and PRTT values were 1.6 ± 0.1 mm/min and 2.4 ± 0.3 mm/15 s in long‐eared hedgehogs and 2.2 ± 0.1 mm/min and 2.5 ± 0.3 mm/15 s in Brandt's hedgehogs, respectively. Mean (SD) Intraocular pressure of right eyes in long‐eared hedgehog and Brandt hedgehog were 19.7 ± 1.4 mmHg and 19.2 ± 2.4 mmHg, respectively. In the left eyes of long‐eared hedgehog and Brandt hedgehog mean (SD) IOP were 19.8 ± 1.5 mmHg and 19.5 ± 2.1 mmHg, respectively. In long‐eared hedgehogs, the most common bacteria were *Staphylococcus epidermidis* and Bacillus spp. In Brandt's hedgehogs, 24 out of 48 eyes had *Staphylococcus epidermidis*, which was the most commonly isolated bacterial species.

**Conclusions:**

This study established reference intervals for IOP, STT and PRTT in hedgehogs and recognised and compared ocular conjunctival microflora in two breeds of hedgehogs.

## Graphical abstract

In this study normal ophthalmic parameters of two breeds of hedgehogs (long eared and brandt hedgehog) has been evaluated, including Iop, Stt, PRTT and conjunctival bacterial and fungal flora.

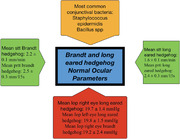

## 1 INTRODUCTION

Hedgehogs are small mammals from the family of Erinaceidae. They are nocturnal animals with protective spines on the back of their body. Seventeen species of hedgehogs are native to different parts of the world, from the Middle East and Central Asia to Australia, Africa and Europe (DK Publishing Inc, 2006). Long‐eared hedgehogs (*Haemiechinus auritus*) are similar to European hedgehogs in appearance with larger ears and coarse fur on their limbs and faces (DK Publishing Inc, 2006). Brandt's hedgehogs (*Paraechinus hypomelas*) are desert‐living hedgehogs with large ears whose size is similar to European hedgehogs (Yusefi et al., 2016).

In a study on 300 hedgehogs in the United Kingdom, cataract was the most common ocular finding. Fifty‐seven out of 300 animals had cataracts, and among them, 54 had nuclear opacities in the lens (Williams et al., 2017). Conjunctivitis, non‐ulcerative keratitis, and uveitis were the other reported common ocular findings (Williams et al., 2017).

Ghaffari et al. (2012) reported normal tear production and intraocular pressure using applanation tonometer in long‐eared hedgehogs. Hedgehogs have gained popularity as exotic pets during the past decades and therefore veterinarians encounter them in the practice. Each species and breeds of animals have their unique physiological and anatomical features. Therefore, due to the increase in the population of hedgehogs as exotic pets during past decades and the high risk of eye injuries, it seems necessary to study the natural structure of the eyes of different species of hedgehogs.

The purpose of this study was to evaluate intraocular pressure, conjunctival bacterial and fungal microflora and measure normal tear production using the Schirmer tear test (STT) and phenol red thread test (PRTT) in two breeds of hedgehogs (long‐eared hedgehogs and Brandt's hedgehogs).

## 2 MATERIALS AND METHODS

The present study was conducted in accordance with the statement of the Association for Research in Vision and Ophthalmology (ARVO) for the use of animals in ophthalmic and vision research.

Forty‐eight hedgehogs from two different breeds, long‐eared hedgehogs (12 males and 12 females) and Brandt's hedgehogs (12 males and 12 females), from a captive rescued colony, were used in this study.

Before the start of the study, all the animals underwent full ophthalmic examination, including slit‐lamp biomicroscopy (Kowa SL‐15; Kowa, Tokyo, Japan), direct (WA11710 Ophthalmoscope, Welch Allyn Inc, NY, USA), and indirect ophthalmoscopy (Binocular Indirect Ophthalmoscope, Welch Allyn Inc, NY, USA), fluorescein staining (Fluorescein Glostrips™, Nomax Inc, St. Louis, USA), STT (Intervet Inc. Merck Animal Health, Summit, NJ, USA) and tonometry (TonoVet^®^, Jorgensen Laboratories, Loveland, CO, USA; ‘d’ setting). In addition to fundus examination with an indirect ophthalmoscope, fundus images were captured from both breeds using an iPhone X and a Pan retinal® 2.2 clear indirect BIO lens (Volk Optical Inc., OH, USA).

Tear production was measured using STT and PRTT (Zone‐Quick®; Menicon America Inc, San Mateo, California, USA) on two separate days. STT strips were placed in the lower conjunctival fornix for 60 s in each eye and the results were recorded as mm/min. Phenol red threads were also placed in the lower conjunctival fornix, and after 15 s the wetted portion of the thread was measured using a ruler and recorded as mm/15 s.

To measure IOP in each eye, a rebound tonometer (TonoVet^®^, Jorgensen Laboratories, Loveland, CO, USA) was used. The device calibration was set to ‘d’.

To perform microbiological sampling one drop of tetracaine (Anestocaine 0.5%, Sinadarou Laboratories Company, Tehran, Iran) was instilled in the eyes. Two sterile microswabs were used to collect samples for the bacterial and fungal cultures. All the microswab samples were transferred in phosphate‐buffered saline (PBS) to the laboratory for culture. Two MacConkey and two blood agar media plates were employed for each eye. One plate of sabouraud dextrose agar (SDA) was used for the fungal culture for each eye. The cultures were examined after 24 h for bacteria, and further isolated cultures were incubated. Standard biochemical tests were performed to identify the isolated organisms.

SPSS statistical software (SPSS Inc., Chicago, IL, USA) was used for the statistical analysis. To test the data normality, a one‐sample Kolmogorov–Smirnov test was used. Descriptive statistical analysis was performed for microbiological data. Mean and standard deviation for the STT, PRTT and IOP data were calculated for each eye. Mean PRTT, STT and IOP were compared between the right and left eyes using a paired‐sample *t*‐test. Pearson's correlation coefficient was used to measure the statistical correlation between STT and PRTT. The *p* value of <0.05 was considered to be statistically significant.

## 3 RESULTS

The mean weight of long‐eared hedgehogs and Brandt's hedgehogs was 465.8 ± 62.5 g and 751.6 ± 37.8 g, respectively. There was a significant difference in the weights of the two breeds (*p* < 0.001).

### 3.1 Tear production

The mean ± SD of STT values in left eyes (OS) and right eyes (OD) was 2.0 ± 0.2 mm/min and 1.8 ± 0.4 m/min in the study population, respectively. No significant difference was found between OS and OD (*p* = 0.5). The mean ± SD of PRTT values in OS and OD was 2.5 ± 0.6 mm/15 s and 2.4 ± 0.4 mm/15 s in hedgehogs, respectively, and there was no significant difference between OS and OD (*p* = 0.5). The mean STT value of OD and OS and the mean PRTT value of OD and OS were computed as the mean STT OU (all eyes) and mean PRTT OU for further analysis.

The mean STT and PRTT values were 1.6 ± 0.1 mm/min and 2.4 ± 0.3 mm/15 s in long‐eared hedgehogs and 2.2 ± 0.1 mm/min and 2.5 ± 0.3 mm/15 s in Brandt's hedgehogs, respectively. The mean STT value of OU was significantly different between two breeds (*p* < 0.001), while the mean PRTT value of OU was not statistically different between two breeds (*p* = 0.4).

The mean ± SD of STT values was identical in male and female hedgehogs (both were 1.9 ± 0.3 mm/min) (*p* = 0.9), while the PRTT values in males (2.3 ± 0.2 mm/15 s) were slightly lower than that in females (2.6 ± 0.3 mm/15 s) (*p* = 0.02).

Pearson's correlation coefficient revealed that there was a positive significant linear correlation between the mean weight and the mean STT value (*r* ^2^ = 0.920, *p* < 0.001), but not between weight and the mean PRTT values (*r* ^2^ = 0.149, *p* = 0.488). There was no correlation between the STT and PRTT values (*r* ^2^ = 0.105, *p* = 0.6).

The intrabreed analysis revealed that there were no significant differences in the mean STT or mean PRTT values between males and females in long‐eared hedgehogs (*p* = 0.8, *p* = 0.3) or Brandt's hedgehogs (p = 0.9, *p* = 0.06) (Table 1).

**TABLE 1:** Mean (SD) of Schirmer tear test (STT) and phenol red thread test (PRTT) in the study population

Parameters	OD	OS	Males	Females	Long‐eared hedgehogs	Brandt's hedgehogs
STT	1.8 (0.4)	2.0 (0.2)	1.9 (0.3)	1.9 (0.3)	1.6 (0.1)	2.2 (0.1)
PRTT	2.4 (0.4)	2.5 (0.6)	2.6 (0.3)	2.3 (0.2)	2.4 (0.3)	2.5 (0.3)

### 3.2 Intraocular pressure

Mean (SD) intraocular pressure of right eyes in long‐eared hedgehog and Brandt hedgehog were 19.7 ± 1.4 mmHg and 19.2 ± 2.4 mmHg, respectively. In left eyes of long‐eared hedgehog and Brandt hedgehog mean (SD) IOP were 19.8 ± 1.5 mmHg and 19.5 ± 2.1 mmHg, respectively. The range of obtained IOP values was 15 to 22 mmHg for both eyes.

There was no significant difference between IOP in right and left eyes (*p* = 0.6). Independent sample *t*‐test revealed no significant difference between males and females in IOP (*p* = 0.8, *p* = 0.6). No significant difference was found between long‐eared hedgehog and Brandt hedgehog in the IOP of right and left eyes (*p* = 0.5, *p* = 0.6). There was no correlation between weight of animals and IOP in the study population (*r* ^2^ = −0.189, *p* = 0.3; *r* ^2^ = −0.117, *p* = 0.5)

### 3.3 Microbiological culture

In long‐eared hedgehogs, the most common bacteria isolated from conjunctiva were *Staphylococcus epidermidis* and *Bacillus* spp. (16 out of 48 eyes: 32% of the total isolated bacteria). *Staphylococcus beta‐haemolytic*, non‐haemolytic *Staphylococcus*, *Streptococcus beta‐haemolytic*, *Corynebacterium* spp., *Moraxella* spp., and *Escherichia coli* were found in 12 (24% of the total isolated bacteria), 6 (12% of the total isolated bacteria), 9 (18% of the total isolated bacteria), 3 (6% of the total isolated bacteria), 3 (6% of the total isolated bacteria), and 1 (2% of the total isolated bacteria) eyes out of 48 eyes, respectively. Only two eyes were negative for bacterial culture (Table 2).

**TABLE 2:** Number of Isolated bacteria and fungi from two breeds of hedgehogs

	Breeds	
Species	Long‐eared	Brandt
Isolated bacteria		
*Staphylococcus epidermidis*	16	24
*Bacillus* spp.	16	14
*Staphylococcus beta‐haemolytic*	12	12
Non‐haemolytic *Staphylococcus*	6	5
*Streptococcus beta‐haemolytic*	9	9
*Corynebacterium* spp.	3	2
*Moraxella* spp.	3	3
*Escherichia coli*	1	2
Isolated fungi		
*Aspergillus* spp.	10	16
*Penicillium* spp.	3	8
*Scopulariopsis* spp.	3	3
Yeast genera	48	42

In the fungal culture, *Aspergillus* spp. was found in 10 out of 48 eyes (15.6% of total isolated fungi). *Penicillium* spp. and *Scopulariopsis* spp. were found in three (4.6% of the total isolated fungi) and three (4.6% of the total isolated fungi) eyes out of 48 eyes, respectively. All the 48 eyes had yeast genera in their fungal culture (75% of total isolated fungi).

In Brandt's hedgehogs, 24 out of 48 eyes had *Staphylococcus epidermidis*, which was the most commonly isolated bacterial species (42.1% of the total isolated bacteria). Fourteen eyes had *Bacillus* spp. (24.5% of the total isolated bacteria), and 12 out of 48 eyes had *Staphylococcus beta‐haemolytic* (21% of the total isolated bacteria). Like long‐eared hedgehogs, nine eyes had *Streptococcus beta‐haemolytic* (15.7% of the total isolated bacteria) while non‐haemolytic *Staphylococcus* was found in five eyes (8.77% of the total isolated bacteria). *Corynebacterium* spp., *Moraxella* spp. and *Escherichia coli* were found in two (3.5% of the total isolated bacteria), three (5.2% of the total isolated bacteria), and two (3.5% of the total isolated bacteria) eyes, respectively. All the eyes were positive for bacterial culture.

Fungal culture revealed 42 eyes positive for yeast genera (60.8% of the total isolated fungi). *Aspergillus* spp. was found in 16 eyes (23.1% of the total isolated fungi), *Penicillium* spp. in 8 eyes (11.5% of the total isolated fungi), and *Scopulariopsis* spp. only in 3 eyes (4.3% of the total isolated fungi) out of 48 eyes in Brandt's hedgehogs. Two eyes were negative for fungal cultures (Table 2).

**FIGURE 1:**
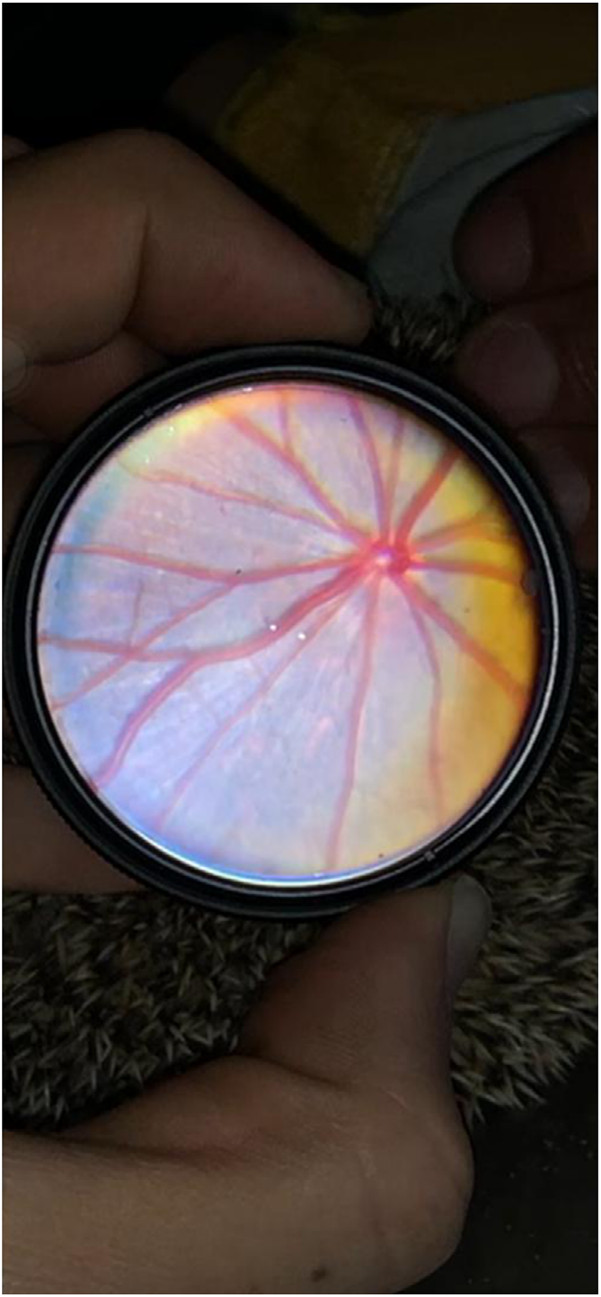
Phoneoscopy of a normal hedgehog fundus

Fundus examination revealed a holangiotic vascular pattern of vessels radiating from the optic disc on an atapetal background. It was the same in both breeds (Figure 1).

## 4 DISCUSSION

The present study reported commensal bacterial and fungal microflora and mean STT, PRTT and IOP in two breeds of hedgehogs.

The tear film is the first refractive surface of the eye. It is vital to health of the eye by protecting and nourishing the cornea (Gelatt, 2020). STT is the conventional method for measurement of tear production. Mean STT has been reported in sedated long‐eared hedgehogs as 1.7 ± 1.2 mm/min (Ghaffari et al., 2012). The mean STT value of long‐eared hedgehogs in our study was 1.6 ± 0.1 mm/min. Brandt's hedgehogs had higher STT values (2.2 ± 0.1 mm/min) than long‐eared hedgehogs in this study.

An alternative method for assessing tear secretion in humans and animals is PRTT. It is more suitable to use in animals with small palpebral fissure lengths like hedgehogs (Rajaei et al., 2013). The mean PRTT value in hedgehogs in this study was about 2.5 mm/15 s, which was lower than mean PRTT values of Syrian hamsters (6.8 mm/15 s) (Rajaei et al., 2013) and rabbits (20.8 mm/15 s) (Biricik et al., 2005).

Results of this study revealed that weight and tear secretion had positive linear correlation in hedgehogs. In contrast, Rajaei et al. (2013) found no correlation between tear production and the weight of the animals in the Syrian hamster. The female hamster has been reported to have higher weight compared to male hamsters (Rajaei et al., 2013).

Intraocular pressure is the balance between secretion and drainage of aqueous humor (AH). AH is secreted by the ciliary body and drained via both conventional and unconventional pathways. An increase and decrease in IOP is usually associated with glaucoma and uveitis respectively (Gelatt, 2020).

Intraocular pressure has been reported in long‐eared hedgehogs using an applanation tonometer under general anaesthesia as 20.1 ± 4 mmHg ranging from 11.5 to 26.5 mmHg (Ghaffari et al., 2012). This study was conducted without general or topical anaesthesia during the time of IOP measurement.

Female hedgehogs have been reported to have higher IOP values than males by means of applanation tonometer (females: 22 mmHg; males: 18 mmHg) (Ghaffari et al., 2012).

In another study, the mean IOP in 10 healthy adult hedgehogs have been reported as 12.6±1.8 mmHg using rebound tonometer (Williams et al., 2017). The Mean IOP in the present study was 19 mmHg and the maximum difference in mean IOP between females and males was 0.4 mmHg, which was not statistically or clinically significant.

The population of commensal bacteria and fungi that interact with each other and immune system are important for regulation of the ocular surface health and prevention of opportunistic pathogens overgrowth (Gelatt, 2020).

In nearly all small mammal and lagomorph species, Gram‐positive bacteria have been reported as the dominant normal conjunctival bacterial flora in health and disease as these bacterial species are found to be normal skin bacteria (Ansari Mood et al., 2019; Cooper et al., 2001; Faghihi et al., 2018; Faghihi & Rajaei, 2019). In rabbits, *Staphylococcus* species were found in nearly 57% of 70 healthy subjects. *Micrococcus* and *Bacillus* spp. were the second and third isolated populations of bacteria in healthy rabbits (Cooper et al., 2001). In Persian squirrels, 83% of the bacterial population was *Staphylococcus* spp. followed by *Corynebacterium* spp. (56%), and *Streptococcus* spp. (53%) as the second and third most common cultivable bacteria (Faghihi et al., 2018). The results of the present study revealed the dominance of *Staphylococcus epidermidis* as a Gram‐positive bacterium.

Gram‐negative bacteria were found in a very small population in the eyes of both long‐eared and Brandt's hedgehogs. *Corynebacterium* spp. was isolated from two hedgehogs in this study*. Bacillus* spp. was common among animals in this study while *Moraxella* spp. and *Escherichia coli* have been isolated in small numbers. *Bacillus* spp. has been found in the eyes of different rodents such as capybara (*Hydrochoerus hydrochaeris*) (Montiani‐Ferreira et al., 2008), chinchilla (*Chinchilla lanigera*) (Lima et al., 2010), opossum (*Didelphis virginiana*) (Pinard et al., 2002), raccoon (*Procyon lotor*) (14), guinea pig (*Cavia porcellus*) (Coster et al., 2008) and prairie dog (*Cynomys ludovicianus*) (Meekins et al., 2015). *Escherichia coli* has been found in Persian squirrels, guinea pigs, prairie dogs, raccoons and chinchillas (Coster et al., 2008; Faghihi et al., 2018; Lima et al., 2010; Meekins et al., 2015; Pinard et al., 2002). *Moraxella* spp. was reported only in rabbits (Cooper et al., 2001). In guinea pigs, population of Gram‐negative bacteria has been reported to increase in guinea pigs with conjunctivitis (Faghihi & Rajaei, 2019).

Many factors have been reported to influence conjunctival microflora, from sampling and culture methods, climates, and habitats to the gender and age of animal species (Gelatt, 2020).

Contact with faeces has been reported to increase Gram‐negative bacteria in the conjunctiva of rodents, especially in captivity (Spinelli et al., 2010). In this study, a few numbers of Gram‐negative bacteria were observed in the bacterial cultures and just one colony of *Escherichia coli* was cultured in an eye. The animals in this study were kept in a captive colony under sanitary conditions, which might be the reason for the low number of faecal Gram‐negative bacteria.

In cats, the number of Gram‐negative bacteria was reported to be increased during cold seasons (Aftab et al., 2019). In clinically normal dogs, the number of positive bacterial cultures was different during cold and warm seasons (Wang et al., 2008). In the present study the sampling was performed in the spring and the animals was housed indoor with constant temperature and humidity.

The effects of gender on bacterial flora in humans have been investigated, and women have been reported to have more cultured bacterial flora and less goblet cell density in alteration of sex hormones. In Persian squirrels (Faghihi et al., 2018), horses (Johns et al., 2011), and pigs (Davidson et al., 1994), the result was the same as humans. In the present study, no significant  differences were observed between the breeds and genders.

Age can influence the immune system and the commensal bacterial population in the body. In humans, a strong correlation has been found between age and conjunctival bacterial flora (Wen et al., 2017). In the present study, the exact age of the animals was unknown.

*Aspergillus* spp., *Penicillium* spp., *Scopulariopsis* spp. and yeast genera were found as fungal conjunctival flora of hedgehogs in our study. *Aspergillus nidulans*, *Cladosporium* spp. and *Aspergillus fumigatus* were isolated from horses with normal eyes (Gemensky‐Metzler et al., 2005). All of the isolated fungi from hedgehogs in this study have been isolated from the conjunctiva of humans with normal eyes (Williamson et al., 1968). *Aspergillus* spp., *Penicillium* spp., *Cladosporium* spp. and *Acremonium* spp. were reported as fungal flora of clinically normal donkeys (Nardoni et al., 2007). The fungal flora of dogs in the south part of the France was same as the flora of hedgehogs in our study (Verneuil et al., 2014). Yeast genera have been isolated from the eyes, ears, and skin of dogs in health and disease (Prado et al., 2008).

There are some limitations in the current study. The first limitation was the environment of the animals. All studied animals were from a colony, and they were not in their natural habitat at the time of this study.

In conclusion, this study established reference intervals for IOP, STT and PRTT in hedgehogs and recognised and compared ocular conjunctival microflora in two breeds of hedgehogs. Future studies on the conjunctival microflora of hedgehogs in health and disease conditions are recommended.

## AUTHOR CONTRIBUTIONS

Ghazal Aftab: Formal analysis, Methodology, Writing – original draft; Farnoosh Arfaee: CRediT contribution not specified; Ahmad Asghari: Visualization; Taghi Zahraei salehi: Investigation.

## CONFLICT OF INTEREST

All authors declare that they have no conflict of interest.

## FUNDING

No specific grants have been received from any financial institutions in the public, commercial or non‐profit sectors.

### ETHICS STATEMENT

All applicable international, national and/or institutional guidelines for the care and use of animals were followed

### PEER REVIEW

The peer review history for this article is available at https://publons.com/publon/10.1002/vms3.1055

## Data Availability

The data that support the findings of this study are available from the corresponding author upon reasonable request.
